# Association between Smoking Habits and Tryptophan Hydroxylase Gene C218A Polymorphism among the Japanese Population

**DOI:** 10.2188/jea.14.94

**Published:** 2005-03-18

**Authors:** Shoichi Mizuno, Hidemi Ito, Nobuyuki Hamajima, Akiko Tamakoshi, Kaoru Hirose, Kazuo Tajima

**Affiliations:** 1 Group of Epidemiology and Health Promotion, Tokyo Metropolitan Institute of Gerontology.; 2 Department of Internal Medicine and Molecular Science, Nagoya City University, Graduate School of Medical Sciences.; 3 Division of Epidemiology and Prevention, Aichi Cancer Center Research Institute.; 4 Department of Preventive Medicine/Biostatistics and Medical Decision Making, Nagoya University Graduate School of Medicine.

**Keywords:** smoking, tryptophan hydroxylase gene, C218A polymorphism, polymerase chain reaction, confronting two-pair primers method

## Abstract

BACKGROUND: Genetic polymorphisms have proposed a new insight in smoking behavior. Genes in serotonin system are one of the candidates because of serotonin’s role in mood regulation. A polymorphism C218A in intron 7 of the *tryptophan hydroxylase* (*TPH*) gene has been hypothesized in relation to smoking predisposition.

METHODS: We examined the association on two Japanese populations: one was from the first-visit outpatients of Aichi Cancer Center Hospital during 3-month period between April and June, 2001 (N=591), and the second was from the examinees who attended a health checkup program supported by the Nagoya municipal government in 2000 (N=446). Written documents on informed consent were obtained and lifestyle questionnaires were recorded. *TPH* C218A genotypes were determined by the polymerase chain reaction with confronting two-pair primers (PCR-CTPP) method.

RESULTS: The frequencies of the *C*- and *A*-allele were 52% and 48%, respectively, which was in Hardy-Weinberg equilibrium. As for current smoking status, no associations were statistically observed. It was, however, indicated that smokers with *A/A* genotype started smoking earlier in their life. Among male health examinees, mean ages at starting smoking were 18.7 (*A/A*), 19.9 (*C/A*), and 22.4 years (*C/C*), (P<0.01). Also, on the Aichi Cancer Center Hospital subjects aged 60 and older, mean ages were 19.0 (*A/A*), 20.2 (*C/A*), and 20.3 years (*C/C*) for males and 22.3 (*A/A*), 31.0 (*C/A*), and 33.0 years (*C/C*) for females (P<0.05).

CONCLUSIONS: The present study demonstrated that the *TPH* C218A polymorphism in intron 7 had no association with current smoking status in Japanese population. The hypothesis of early smoking initiation of *A/A* genotype was partially in agreement.

Smoking is known to be a major health hazard. While its prevalence continues to decline in middle-aged and older Japanese males,^1^ it is still high among developed countries. The Health Promotion Law in 2003 is now in effect in Japan, and it is expected that the incidence of smoking will decrease. The prevalence of smoking is relatively high for the generations who were born after World War II^2^ and according to a recent study, juveniles start smoking is at much younger age.^3^

The associations of genetic polymorphisms and smoking suggested a new insight.^4^^,^^5^ Genes related to dopaminergic reward pathways are major targets and their modifiers,^6^^-^^8^ including serotonin, are all reasonable candidates. Along with these lines, because the *tryptophan hydroxylase (TPH)* gene codes for a primary enzyme in the biosynthesis of serotonin and its genetic variation were able to alter the risk for smoking dependence, a polymorphism *TPH* C218A in a non-coding region, intron 7, linked to C779A variant,^9^^-^^10^ have been studied and hypothesized that it might play a role in smoking predisposition, smoking initiation and/or persistence.^11^^,^^12^

To investigate the role of the *TPH* C218A polymorphism in the smoking behavior for the Japanese, we conducted an epidemiologic population study.

## METHODS

### Study subjects

There were two samples in this study. One was sampled from first-visit out-patients at Aichi Cancer Center during a 3-month period between April and June, 2001 on a framework of the Hospital-based Epidemiologic Research Program at Aichi Cancer Center-II (HERPACC-II).^13^ Among 1,022 first-visit outpatients, 595 provided a 7 mL of peripheral blood. For each patient, a written form of informed consent was obtained for gene polymorphism tests without specification of names of polymorphisms and then consecutively invited to lifestyle questionnaires and peripheral blood donation. Excluding four individuals for whom genotypes could not be identified, the number of study subjects was 591 in total, with 267 males and 324 females. In Aichi Cancer Center series, never smokers were identified as those who smoked less than 100 cigarettes in the past, current smokers as those who smoked at least in the past one year, and former smokers as those who quit smoking more than one year before the questionnaire study. This study had been approved by the Ethical Committee at Aichi Cancer Center before the study started (Ethical Committee Approval Numbers 41-2).

The second series of subjects were sampled from the examinees in August and September in the year 2000, who attended a health checkup community program for the inhabitants of Nagoya City supported by the Nagoya Municipal Government. A written informed consent to anonymous use of the residual blood as well as information on demographic characteristics and smoking habits was obtained after the blood draw for the health checkup. Usually, about 2 mL of blood was left after the routine tests. No extra blood draw was conducted. Out of 489 examinees invited to the study, 468 (96%) agreed to provide their residual blood for genetic tests along with the requested information. Three residual blood samples did not allow DNA extraction. Eleven participants with a cancer history, including two individuals with stomach cancer, were excluded. Within the remaining 454 subjects (126 males and 328 females) aged 35 to 85 years, genotypes of three males and five females could not be identified so that the rest of 123 males and 323 females were the subjects of this study. The Ethical Committee of Aichi Cancer Center had approved this study in 1999 (Approval number 12-23).

### Genotyping

DNA was extracted from 200 µL buffy coat preserved at -80°C by QIAamp DNA Blood Mini Kit (QIAGEN Inc., Valencia, CA). The genotyping was conducted by a novel PCR technique, polymerase chain reaction with confronting two-pair primers (PCR-CTPP).^14^^,^^15^ The primers were F1: 5′ATC CCT TCT ATA CCC CAG A, R1: 5′CTA TGC TCA GAA TAG CAG CTA T, F2: 5′TAA TTG ACA ACC TAT TAG GTG C, and R2: 5′TAC CTA TGG ACA TCC ACA TG for detecting C218A polymorphism. The underlined are the site of a single nucleotide polymorphism.

Genomic DNA (30ng to 100ng) was used in a volume of 25µL with 0.1mmol dNTPs, 25 pmol of each primer, 0.5 units of “AmpliTaq Gold” (Perkin-Elmer Corp., Foster City, CA, USA), and 2.5 µL 10× PCR Buffer including 15mmol MgCl_2_. A 2.5µL of glycerol was added in genotyping for C218A polymorphism. PCR for C218A was conducted as follows; a 10 min of initial denature at 95°C, 35 cycles of 1 min at 95°C, 1 min at 60°C, and 1 min 72°C, and a 5 min of final extension at 72°C.

**Figure 1.**
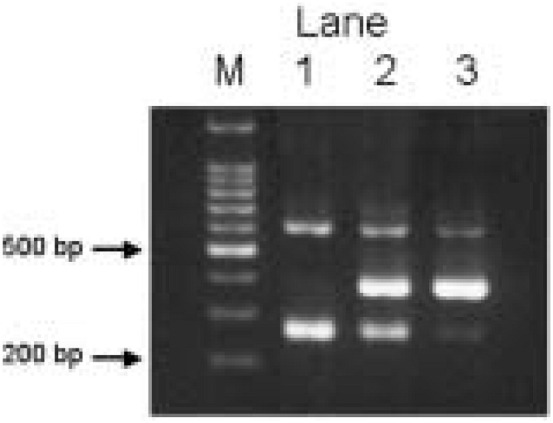
Representative examples of agarose gel electrophoresis.

Amplified PCR products were visualized on a 2% agarose gel with ethidium bromide staining. Genotyping of C218A is 382-bp for C allele and 265-bp for *A* allele with a 603-bp common band. Figure 1 shows a representative result of genotyping for C218A.

### Statistical analysis

C218A polymorphism was analyzed according to smoking status. Frequencies were tested statistically using chi-square tests. Among current and/or ever smokers, the number of cigarettes smoked per day, ages at starting smoking, and scores of Fagerstöm Test for Nicotine Dependency^16^ were analyzed by using an analysis of variance, a multivariate analysis, and/or a nonparametric test. The fitness for Hardy-Weinberg equilibrium was examined by “genhwi” command of the computer program STATA^®^ Version 7 (STATA Corporation, College Station, TX, USA).

C218A polymorphism, lane M for a 100-bp ladder marker, lane 1 for *A/A* genotype, lane 2 for *C/A* genotype, and lane 3 for *C/C* genotype.

## RESULTS

Ages of Aichi Cancer Center subjects were 56.8±12.7 (mean±standard deviation) years in males (N=267), and 49.9±13.9 years in females (N=324). Ages of health check-up examinees were 63.7±11.1 years in males (N=123), and 56.3±11.7 in females (N=323). Aichi Cancer Center subjects were younger in age than health check-up examinees.

Table 1 shows the numbers of study subjects by smoking status according to sex and age. Among the Aichi Cancer Center subjects, prevalence of smoking status was as follows: current, former, and never smoker was 41.6%, 38.2%, and 20.2% in males, and 17.9%, 7.1%, and 74.4% in females. Two subjects (0.6%) had no smoking information and they were omitted in subsequent analysis. For the health check-up examinees, the smoking prevalence was; 37.4%, 4.9%, and 57.7% in males, and 5.6%, 1.2%, and 93.2% in females.

**Table 1.** Sex and age distributions of the subjects according to smoking status.

Age(year)	Male					Female						

	Current	Former	Never	Total		Current	Former		Never		Unknown	Total
Outpatients at Aichi Cancer Center Hospital												
18-29	5	2	2		9	8		1		13	2	24
30-39	11	3	6		20	19		3		43		65
40-49	18	10	5		33	15		3		40		58
50-59	41	31	14		86	12		8		74		94
60-69	26	33	16		75	4		7		42		53
70-79	10	23	11		44	0		1		29		30

Total	111	102	54		267	58		23		241		324

Health Examiniees												
35-39	7	0	2		9	3		1		35		39
40-49	4	0	3		7	8		1		41		50
50-59	4	0	6		10	5		2		83		90
60-69	20	3	39		62	1		0		109		110
70-79	11	2	18		31	1		0		29		30
80-85	0	1	3		4	0		0		4		4

Total	46	6	71		123	18		4		301		323

Table 2 shows C218A genotype frequency by sex. Difference was not statistically significant between males and females. Genotype frequency was in excellent agreement with Hardy-Weinberg equilibrium for both males and females. In a total of 1037 subjects examined, genotype frequency of *C/C*, *C/A*, and *A/A* was 27.6%, 48.1%, and 24.3%. The overall frequency of *C*- and A-allele was 51.6% and 48.4%, respectively.

**Table 2.** *TPH* C218A polymorphism by sex.

	*C/C*	*C/A*	*A/A*	Total
Aichi Cancer Center Outpatients				
Male	70(26.2)	129(48.3)	68(25.5)	267
Female	84(25.9)	162(50.0)	78(24.1)	324
Total	154(26.1)	291(49.2)	146(24.7)	591
Health Examinees				
Male	35(28.5)	59(48.0)	29(23.6)	123
Female	97(30.0)	149(46.1)	77(23.8)	323
Total	132(29.6)	208(46.6)	106(23.8)	446

Total	286(27.6)	499(48.1)	252(24.3)	1037

Percentages in parentheses

Table 3 shows C218A genotype frequency according to smoking status. Association was not statistically significant.

**Table 3.** *TPH* C218A polymorphism by smoking status.

Smoking Status	Male				Female			
	
	*C/C*	*C/A*	*A/A*	Total	*C/C*	*C/A*	*A/A*	Total
ACC Outpatients								
Current	30(27.0)	52(46.8)	29(26.1)	111	17(29.3)	24(41.4)	17(29.3)	58
Former	27(26.5)	51(50.0)	24(23.5)	102	3(13.0)	15(65.2)	5(21.7)	23
Never	13(24.1)	26(48.1)	15(27.8)	54	64(26.6)	122(50.6)	55(22.8)	241
Total	70(26.2)	129(48.3)	68(25.5)	267	84(26.1)	161(50.0)	77(23.9)	322

Pearson chi-square = 0.52, p> 0.97 for males,					Pearson chi-square =4.57, p>0.33 for females.			

Health Examinees								
Current	12(26.1)	23(50.0)	11(23.9)	46	6(33.3)	8(44.4)	4(22.2)	18
Former	0( 0.0)	4(66.7)	2(33.3)	6	1(25.0)	2(50.0)	1(25.0)	4
Never	17(23.9)	32(45.1)	22(31.0)	71	70(23.3)	139(46.2)	92(30.6)	301
Total	29(23.6)	59(48.0)	35(28.5)	123	77(23.8)	149(46.1)	97(30.0)	323

Pearson chi-square = 2.69, p> 0.61 for male,					Pearson chi-sqaure =1.17, p>0.88 for females.			

Percentages in parentheses.

Among ever smokers with information obtained, ages at starting smoking, the number of cigarettes smoked per day, Fagerstöm test for nicotine dependence were analyzed. As shown in Table 4, C218A polymorphism did not show statistical difference among Aichi Cancer Center subjects. As for the male health examinees, however, the subjects of *A/A* genotype started smoking earlier in their life, 18.7 years, compared to *C/A* genotype, 19.9 years, and *C/C* genotype, 22.4 years. The variation was statistically significant (p<0.01), and a test for linear trend showed a significance (p<0.01).

**Table 4.** Age at starting smoking by *TPH* C218A polymorphism.


	Aichi Cancer Center Outpatients				Health Examinees			

	Male		Female		Male		Female	
	
	Mean(SD)	N	Mean(SD)	N	Mean(SD)	N	Mean(SD)	N

*C/C*	19.5(2.8)	58	24.5(8.3)	19	22.4(4.5)	12	21.3( 2.5)	4
*C/A*	19.7(2.1)	103	23.2(7.0)	38	19.9(1.5)	27	27.4(12.4)	10
*A/A*	19.7(2.7)	53	26.0(9.2)	23	18.7(2.0)	11	24.0( 4.7)	7

	F=0.14, p>0.87,		F=0.95, p>0.39,		F=6.23, p<0.01,		F=0.70, p>0.50	

SD: standard deviation

As for Aichi Cancer Center subjects with ages 60 and older, the subjects with genotype *A/A* started smoking earlier than the subjects of genotype *C/A* or *C/C*, which was statistically significant (p<0.05) from a multivariate regression analysis with sex adjusted (Table 5).

**Table 5.** Age at starting smoking of Aichi Cancer Center outpatients with ages 60 and older.

	Male		Female	
	
	Mean(SD)	N	Mean(SD)	N
*C/C*	20.3(3.4)	26	33.0(13.2)	3
*C/A*	20.2(2.6)	47	31.0( 8.1)	5
*A/A*	19.0(1.1)	20	22.3( 2.5)	3

p<0.05, (A/A started smoking earlier than other genotypes),

P-value was obtained by a multivariate regression analysis with sex adjusted.

SD: standard deviation

Percentages in parentheses.

## DISCUSSION

We have been investigated genetic influence on one’s smoking behavior, on genes related to neurotransmitters of dopaminergic reward pathways^17^^-^^19^ and inflammation.^20^ The present study was conducted because the *TPH* gene codes for a primary enzyme in the biosynthesis of serotonin, and its genetic variation was meaningfully able to alter the risk for smoking dependence, and two closely linked variants in intron 7 have been studied extensively.^5^^,^^9^^-^^12^

The present study demonstrated that the C218A polymorphism in intron 7 of the *TPH* gene showed no significant association on the Japanese population with respect to their current smoking status. Smokers with genotype *A/A*, however, might be partially predisposed to early smoking initiation.

Overall frequencies of the TPH C218A *C*- and A- allele were 52% and 48% for our Japanese study population (N=1037). Sullivan et al. investigated the C218A polymorphism among regular smokers (N=482) and lifetime nonsmokers (N=207), and reported *A*- allele frequencies of 43.0% and 31.1%, respectively, recognizing a statistical significance.^12^ Lerman et al., however, reported an *A*- allele frequency of 43% among smokers and 40% among nonsmokers, negating any statistical association on smoking status.^11^ As shown in Table 3, the C218A polymorphism showed no association with the current smoking status in Japanese population.

Analyzing characteristics of smokers, aforementioned researchers, Lerman et al.,^11^ found that the ages when they started smoking were 15.6, 17.1, and 17.4 years, according to genotypes of *A/A*, *A/C* and *C/C*, respectively, and concluded that the presence of an *A/A* genotype may predispose a person to initiate smoking early. Compared to those with other genotypes, the individuals with the *A/A* genotype were expected to exhibit their predisposition for smoking by taking up smoking about 1.5 years earlier than the others. As shown in Tables 4 and 5, our results were in agreement only in part. Among the male health check-up examinees, the data on those with the *A/A* genotype who had ever smoked showed a tendency to initiate smoking early (*i.e.*, a mean of 2.0 years earlier than other genotypes). The extent of earliness in smoking initiation, 2.0 years, was in good agreement to the finding of Lerman et al. Since the ages of Aichi Cancer Center subjects were younger, we conducted a subpopulation analysis, restricting the Aichi Cancer Center study subjects to those aged 60 and older (as shown in Table 5) with consideration that smoking behavior is much age dependent. Then, smokers of the *A/A* genotype started smoking earlier than smokers of other genotypes (p<0.05), which was demonstrated in a gender-adjusted multivariate regression analysis.

Considering the discrepancy observed in the smoking history of the Aichi Cancer Center subjects, especially ages when smoking was started, those who ever smoked at all were further tabulated in Table 6 among the Aichi Cancer Center subjects with their ages restricted to less than fifty. The association with a C218A polymorphism was demonstrated on neither the age when smoking was started, the daily number of cigarettes, nor Fagerstöm Test Score for Nicotine Dependency. The age when smoking was started was much lower, especially in women.

**Table 6.** Analyses of smoking history by *TPH* C218A polymorphism (mean and standard deviation).

Smoking variables	*A/A*	*C/A*	*C/C*	F statistic	p value
Males (N)	9	24	16		
Age at starting smoking (year)	18.7±1.7	19.1±1.8	19.9±3.8	0.82	0.45
Nicotine dependence (FTND)	3.5±3.1	3.9±1.9	4.3±2.9	0.32	0.73
Smoking rate(cigarettes/day)	17.0±9.7	20.7±8.1	23.4±11.5	1.3	0.29
Females (N)	12	25	12		
Age at starting smoking (year)	20.2±4.2	20.6±3.0	21.9±7.5	0.45	0.64
Nicotine dependence (FTND)	3.3±2.7	3.2±2.0	2.3±2.6	0.65	0.53
Smoking rate(cigarettes/day)	14.9±13.4	13.1±7.3	11.2±5.5	0.54	0.59

The Aichi Cancer Center outpatients with ages younger than 50 years were analyzed.

FTND: Fagerstöm Test for Nicotine Dependency

While the tendency to smoke is declining in Japan, the age at which youngsters take up smoking has shown a marked increase in recent years. It is believed that the trend has its origin in socio-environmental factors. The admittance of the genetic factor described above on a middle-to- aged population might be due to the results obtained from smoking cessation after middle ages. Individuals start smoking for various reasons, and the factors in the background might sometime act multiplicatively or additively. In the future when the prevalence of smoking stabilizes, the contribution of genetic factors may be much more easily evaluated and our results may gain significance.

Although our results were not altogether convincing for the younger generation, the hypothesis that an individual with the *A/A* genotype initiates smoking early in life was partially in agreement with the data obtained from the Japanese population. This finding together with other works provides preliminary knowledge in understanding the role of serotonergic genes on smoking behavior. Research on genes and/or socio-behavioral-environmental factors and their interactions is encouraged to obtain a better understanding of the prevalence of smoking and its future control among the Japanese people.

## References

[1] Japan Tobacco Industry, Inc. Surveys on smoking rates in Japan, 1965-2000. (See also, Japan Health Promotion and Fitness Foundation: Available from URL: www.health-net.or.jp/tabacco/)

[2] Research Group on Evaluation of Risk Factors for Cancer by Large-Scale Cohort Study (Chairman, Aoki K, 1988-1995). Baseline results of a large-scale cohort study on evaluation of risk factors on cancer: Findings obtained from a questionnaire survey by item, sex and age-group. Ministry of Education, Science and Culture, Tokyo. Table 10 (Smoking habits). 1996:36.

[3] Osaki Y, Minowa M, Suzuki K, Wada K. Cigarette smoking among junior and senior high school students in Japan: report of 1996. Kousei No Shihyo 1999;46(11):16-22.

[4] Batra V, Patkar AA, Berrettini WH, Weinstein SP, Leone FT. The genetic determinants of smoking. Chest 2003;123:1730-9. 12740294 10.1378/chest.123.5.1730

[5] Tyndale RF. Genetics of alcohol and tobacco use in humans. Ann Med 2003;35:94-121. 12795339 10.1080/07853890310010014

[6] Comings DE, Blum K. Reward deficiency syndrome: genetic aspects of behavioral disorders. Prog Brain Res 2000;126:325-41. 11105655 10.1016/S0079-6123(00)26022-6

[7] Noble EP. D2 dopamine receptor gene in psychiatric and neurologic disorders and its phenotypes. Am J Med Genet 2003;116B:103-25. 12497624 10.1002/ajmg.b.10005

[8] Ueno S. Genetic polymorphisms of serotonin and dopamine transporters in mental disorders. J Med Invest 2003;50:25-31. 12630565

[9] Nielsen DA, Jenkins GL, Stefanisko KM, Jefferson KK, Goldman D. Sequence, splice site and population frequency distribution analyses of the polymorphic human tryptophan hydroxylase intron 7. Brain Res Mol Brain Res 1997;45:145-8. 9105682 10.1016/s0169-328x(96)00304-x

[10] Paoloni-Giacobino A, Mouthon D, Lambercy C, Vessaz M, Coutant-Zimmerli S, Rudolph W, . Identification and analysis of new sequence variants in the human tryptophan hydroxylase (TpH) gene. Mol Psychiatr 2000;5:49-55. 10.1038/sj.mp.4000647 10673768

[11] Lerman C, Caporaso NE, Bush A, Zheng YL, Audrain J, Main D, . Tryptophan hydroxylase gene variant and smoking behavior. Am J Med Gene 2001;105:518-20. 10.1002/ajmg.1476 11496367

[12] Sullivan PF, Jiang Y, Neale MC, Kendler KS, Straub RE. Association of the tryptophan hydroxylase gene with smoking initiation but not progression to nicotine dependence. Am J Med Genet 2001;105:479-84. 11449402 10.1002/ajmg.1433

[13] Hamajima N, Matsuo K, Saito T, Hirose K, Inoue M, Takezaki T, . Gene-environment interactions and polymorphism studies of cancer risk in the hospital-based epidemiologic research program at Aichi Cancer Center II (HERPACC-II). Asian Pac J Cancer Prev 2001;2:99-107. 12718640

[14] Hamajima N, Saito T, Matsuo K, Kozaki K, Takahashi T, Tajima K. Polymerase chain reaction with confronting two-pair primers for polymorphism genotyping. Jpn J Cancer Res 2000;91:865-8. 11011111 10.1111/j.1349-7006.2000.tb01026.x PMC5926438

[15] Hamajima N, Saito T, Matsuo K, Tajima N. Competitive amplification and unspecific amplification in polymerase chain reaction with confronting two-pair primers. J Mol Diagn 2002;4:103-7. 11986401 10.1016/S1525-1578(10)60688-5 PMC1906991

[16] Heatherton TF, Kozlowski LT, Frecker RC, Fagerstrom KO. The Fagerstrom Test for Nicotine Dependence: a revision of the Fagerstrom Tolerance Questionnaire. Br J Addict 1991;86:1119-27. 1932883 10.1111/j.1360-0443.1991.tb01879.x

[17] Yoshida K, Hamajima N, Kozaki Ki, Saito H, Maeno K, Sugiura T, . Association between the dopamine D2 receptor *A2/A2* genotype and smoking behavior in the Japanese. Cancer Epidemiol Biomarkers Prev 2001;10:403-5. 11319183

[18] Ito H, Hamajima N, Matsuo K, Okuma K, Sato S, Ueda R, . Monoamine oxidase polymorphisms and smoking behaviour in Japanese. Pharmacogenetics 2003;13:73-9 12563176 10.1097/00008571-200302000-00003

[19] Hamajima N, Ito H, Matsuo K, Saito T, Tajima K, Ando M, . Association between smoking habits and dopamine receptor D2 *taq*I A *A2* allele in Japanese males: a confirmatory study. J Epidemiol 2002;12:297-304. 12395869 10.2188/jea.12.297 PMC10616368

[20] Hamajima N, Katsuda N, Matsuo K, Saito T, Ito LS, Ando M, . Smoking habit and interleukin *1B C-31T* polymorphism. J Epidemiol 2001;11:120-5. 11434423 10.2188/jea.11.120 PMC11701264

